# Whole-exome sequencing identifies a homozygous pathogenic variant in *TAT* in a girl with palmoplantar keratoderma

**DOI:** 10.1016/j.ymgmr.2019.100534

**Published:** 2019-11-22

**Authors:** Fady Hannah-Shmouni, Lauren MacNeil, Irene Lara-Corrales, Elena Pope, Peter Kannu, Neal Sondheimer

**Affiliations:** a Section on Endocrinology and Genetics, *Eunice Kennedy Shriver* National Institute of Child Health and Human Development, National Institutes of Health, Bethesda, MD 20892, USA; b Division of Pediatric Laboratory Medicine, The Hospital for Sick Children, Toronto, Ontario, Canada; c Department of Laboratory Medicine and Pathobiology, University of Toronto, Toronto, Ontario, Canada; d Pediatric Dermatology, The Hospital for Sick Children, University of Toronto, Canada; e Clinical Genetics, Division of Clinical and Metabolic Genetics, The Hospital for Sick Children, University of Toronto, Canada

**Keywords:** PPK, Palmoplantar keratoderma

## Abstract

Palmoplantar keratoderma (PPK) is a defect in cornification that is characterized by progressive hyperkeratosis of palms and soles. Many phenotypes are linked with PPK, making exome-based diagnosis increasingly efficient. In this report, we identified tyrosinemia type II on whole-exome sequencing in a 7-year-old Syrian refugee that presented with PPK. Dietary therapy helped improve her overall symptoms.

## 1 Background

Palmoplantar keratoderma (PPK) is a defect in cornification that is characterized by progressive hyperkeratosis of palms and soles. PPK can be either acquired through environmental exposure or infection or may be the consequence of an inherited disorder (Table 1) [1]. Many phenotypes are linked with PPK (Table 1) [1], making exome-based diagnosis increasingly efficient, particularly if the phenotype is overlapping or not straightforward.

**Table 1:** The major hereditary palmoplantar keratodermas (PPK).

Palmoplantar keratoderma	Gene	Inheritance
Striate		
• Type I	*DSG1*	AD
• Type II	*DSP*	AD

Diffuse		
• Epidermolytic, Vörner type	*KRT1*	AD
• Epidermolytic of Greither	*KRT1*	AD
• Diffuse nonepidermolytic	*KRT1*	AD
• Gamborg-Nielsen	*SLURP1*	AR
• Diffuse, Bothnian type	*AQP5*	
• Diffuse, Nagashima type	*SERPINB7*	AR
• Sclerotylosis, Huriez syndrome	*SMARCAD1*	AD
• *Keratolytic winter erythema (*Oudtshoorn skin disease)	*CTSB*	AD
Focal	*KRT6C* *KRT16*	AD

Punctate		
• Type I	*AAGAB*	AD
• Type II	?	AD
• Type III	?	AD
• Focal acral hyperkeratosis	?	AD
• Hereditary papulotranslucent acrokeratoderma	?	

Complex		
• Loricrin keratoderma	*LOR*	AD
• Olmsted syndrome	*TRPV3, MBTPS2, PERP*	AD, AR, XL
• Striate with wooly hair	*KANK2*	AR
• Palmoplantar keratoderma with congenital alopecia	*GJA1*	AD
• Dyschromatosis universalis hereditaria	*SASH1*	AD
• Tyrosinemia type 2	*TAT*	AR

Abbreviations: AD, autosomal dominant; AR, autosomal recessive; XL, X-linked.

Adapted from Guerra et al. [1].

### 1.1 Case presentation

**Fig. 1:**
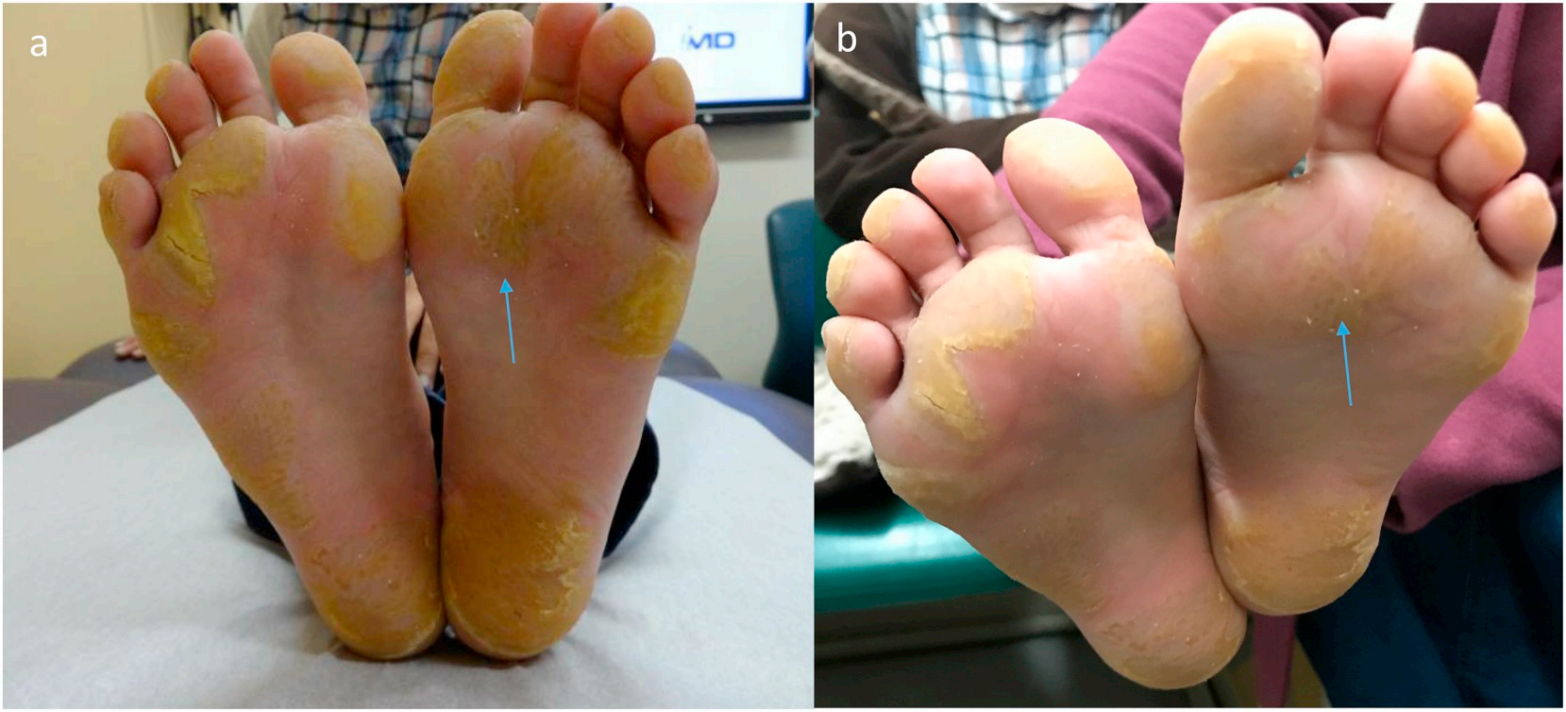
Palmoplantar keratoderma on the patient's soles pre (a) and post (b) dietary modifications with minor improvement in keratoderma (arrows).

A 7-year-old Syrian refugee presented to the genodermatosis clinic following several years of progressive painful and yellowish thickening of the palms and soles, localized mostly to pressure points (Fig. 1). She also had photophobia, poor vision and ocular pain on the left eye, developmental delay, and difficulty with her learning and understanding instructions. Parents reported that these problems had started at age 4, had been progressive and now were affecting her daily activities and disrupting sleep. Topical keratolytics had been used without success. Physical examination revealed a focal palmoplantar keratoderma with no dysmorphic features (Fig. 1).

**Fig. 2:**
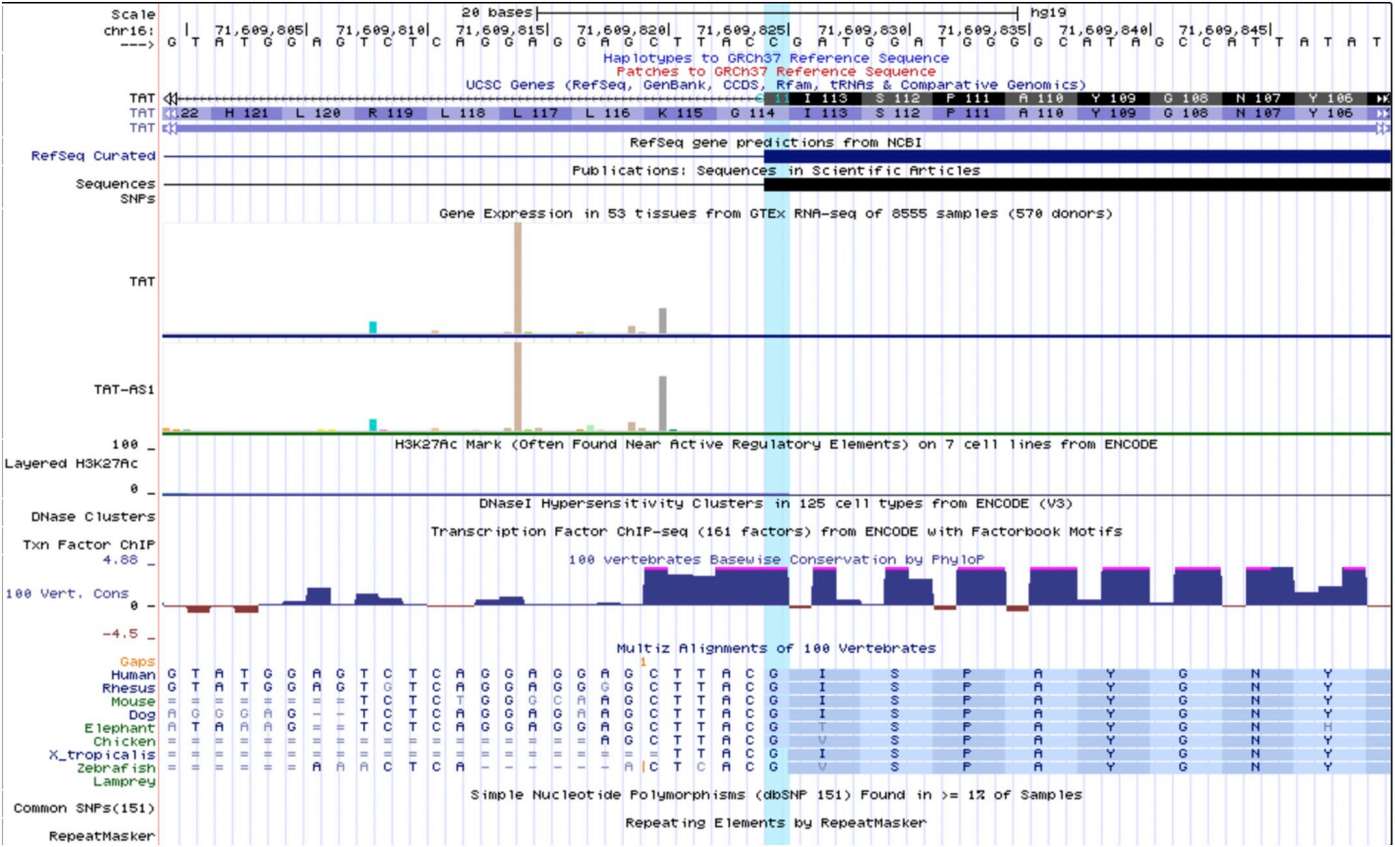
The homozygous pathogenic variant in exon 3 of *TAT* (c.340G > A, p.Gly114Ser; NM_000353.2) occurs at a position that is conserved across all species (Figure source: UCSC Genome Browser).

Her parents are first cousins and otherwise healthy. Her brother shared similar clinical features. Thus, the possibility of an underlying autosomal recessive condition was considered. Genetic testing for PPK using Exome/Slice (phenotype directed exome testing) showed a novel homozygous likely pathogenic missense variant in exon 3 of *TAT* (c.340G > A, p.Gly114Ser; NM_000353.2, rs759311161), consistent with tyrosinemia type II. This variant was not observed in approximately 6500 individuals of European and African American ancestry in the NHLBI Exome Sequencing Project, and rarely reported, only at the heterozygous state, in ExAC or GnomAD, in keeping with not being a benign variant. This substitution occurs at a position that is conserved across all species, and not likely to impact protein structure (Fig. 2). This variant is predicted as disease causing by MutationTaster, Provean and SIFT. Other reported disease causing variants in *TAT* that support the causality of this variant include c.355C > T (p.Arg119Trp; NM_000353) [2] and c.341G > C (p.Gly114Ala; NM_000353.3) [3]. Her brother was confirmed to have the same genetic alteration via Sanger sequencing.

Plasma amino acid demonstrated hypertyrosinemia (1191 μmol/L, reference interval: 45–126 μmol/L) with normal phenylalanine levels. Urine organic acid showed the presence of tyrosine metabolites, including L-form of 4-hydroxyphenyllactate, 4-hydroxyphenylacetate, and 4-hydroxyphenylpyruvate, and small quantities of *N*-acetyltyrosine and 4-tyramine. She was started on dietary restriction of phenylalanine and tyrosine with clinical improvement of her symptoms (Fig. 1).

The clinical and laboratory findings in this patient are consistent with a diagnosis of Tyrosinemia type II (OMIM #276600), previously referred to as oculocutaneous tyrosinemia or Richner–Hanhart syndrome [4]. Tyrosinemia type 2 is an autosomal recessive disorder characterized by hypertyrosinemia on plasma amino acids (typically >500 μmol/L), late-onset PPK, developmental delay, variable intellectual disability, and herpetiform corneal ulcers. Patients may present with either ocular or skin findings in the first decade of life [5]. PPK is a major feature of Tyrosinemia type II, typically manifesting as focal lesions in the palms and soles that usually develop after the first year of life, although may appear earlier or even later (Table 1) [5,6]. Tyrosinemias are identified on newborn screening. However, our patient was born in Syria, a country without a newborn screening program, hence the delay in diagnosis. This case illustrates the utility of phenotype-directed exome testing, particularly in diagnoses like PPK where there are many genotypes that are linked with a common phenotype (Table 1). It also demonstrated the importance of taking a complete history and thinking beyond the skin in pediatric dermatology patients.

Tyrosinemia type II is is caused by disease causing variants in the *TAT* gene (16q22.2, OMIM #613018) [7], which encodes tyrosine aminotransferase, the first enzyme involved in the degradation of tyrosine [4]. This enzyme catalyzes the transamination reaction that converts tyrosine to *p*-hydroxyphenylpyruvate in the liver. The inability to properly deaminate tyrosine in the cytosol of hepatocytes via *TAT* results in the release into circulation and subsequent hypertyrosinemia. Excess tyrosine is metabolized by other enzymes, resulting in additional metabolites used to support the diagnosis of tyrosinemia. Functional mitochondrial tyrosine aminotransferase in tissues without the full tyrosine metabolic pathway produces the 4-hydroxyphenylketones detected in urine. Additionally, acetylation or oxidation produces *N*-acetyltyrosine and 4-tyramine when tyrosine concentrations are high. In this case, the patient presented with typical symptoms of tyrosinemia type II, but the diagnosis was made through genetic testing. Treatment using protein restriction and supplementation of amino acids without phenylalanine and tyrosine have been shown to improve symptoms [4,5].

The patient was initiated on dietary restriction of phenylalanine and tyrosine with minor improvement in symptoms following 3 months of dietary changes (Fig. 1, arrows) [4,8]. She was seen in the dermatology clinic and started on 20% salicylic acid in petrolatum base on her skin lesions with nightly occlusions. An ophthalmological examination revealed left corneal ulcerations. Our case highlights the potential value of using genetic testing to diagnose skin findings of rare metabolic diseases.

## Consent

Written informed consent was obtained from the parents of the patient for the publication of this case report and any potentially-identifying information/images.
